# Preparation of β-Cyclodextrin Functionalized Platform for Monitoring Changes in Potassium Content in Perspiration

**DOI:** 10.3390/molecules28197000

**Published:** 2023-10-09

**Authors:** Ruixiang Liu, Xiaofeng Shi

**Affiliations:** College of Physical Education, Shanxi University, Taiyuan 030006, China; shixf@sxu.edu.cn

**Keywords:** potassium, sweat monitoring, wearable sensor, reduced graphene oxide, screen printing, ion-selective electrode

## Abstract

The monitoring of potassium ion (K^+^) levels in human sweat can provide valuable insights into electrolyte balance and muscle fatigue non-invasively. However, existing laboratory techniques for sweat testing are complex, while wearable sensors face limitations like drift, fouling and interference from ions such as Na^+^. This work develops printed electrodes using β-cyclodextrin functionalized reduced graphene oxide (β-CD-RGO) for selective K^+^ quantification in sweat. The β-CD prevents the aggregation of RGO sheets while also providing selective binding sites for K^+^ capture. Electrodes were fabricated by screen printing the β-CD-RGO ink onto conductive carbon substrates. Material characterization confirmed the successful functionalization of RGO with β-CD. Cyclic voltammetry (CV) showed enhanced electrochemical behavior for β-CD-RGO-printed electrodes compared with bare carbon and RGO. Sensor optimization resulted in a formulation with 30% β-CD-RGO loading. The printed electrodes were drop-casted with an ion-selective polyvinyl chloride (PVC) membrane. A linear range from 10 μM to 100 mM was obtained along with a sensitivity of 54.7 mV/decade. The sensor showed good reproducibility over 10 cycles in 10 mM KCl. Minimal interference from 100 mM Na^+^ and other common sweat constituents validated the sensor’s selectivity. On-body trials were performed by mounting the printed electrodes on human subjects during exercise. The K^+^ levels measured in sweat were found to correlate well with serum analysis, demonstrating the sensor’s ability for non-invasive electrolyte monitoring. Overall, the facile synthesis of stable β-CD-RGO inks enables the scalable fabrication of wearable sensors for sweat potassium detection.

## 1. Introduction

The monitoring of electrolytes and metabolites in human sweat has garnered substantial interest in recent years for non-invasive health and fitness monitoring. Unlike blood and interstitial fluid, which require invasive sampling, sweat can be collected conveniently on the skin’s surface, making it well-suited for continuous monitoring applications [1,2]. The concentrations of various ions and small molecules in sweat have been shown to provide meaningful physiological information, such as electrolyte imbalance, cystic fibrosis, dehydration and muscle fatigue [3]. Among the various constituents of sweat, the monitoring of potassium ion (K^+^) levels is of particular diagnostic value.

K^+^ is one of the most abundant cations in the human body, playing a vital role in cell membrane potential, protein synthesis and electrical conductivity in nerves and muscles [4]. The normal concentration range of K^+^ in sweat is typically between 5 to 15 mM. Elevated K^+^ levels in sweat, termed hyperkalemia, can be indicative of acute kidney injury, adrenal insufficiency, tissue damage or excessive intake [5]. On the other hand, low K^+^ levels or hypokalemia may occur due to kidney disorders, malnutrition, diarrhea or the use of diuretics [6]. Since sweat K^+^ directly correlates with serum K^+^, its real-time monitoring can provide prompt insights into electrolyte balance non-invasively. This could be invaluable for applications ranging from athletic monitoring to critical care.

However, sweat-based analysis poses inherent challenges as well. Sweat exhibits complex kinetics across the skin surface, with spatial variations in sweat rate, flow rate and analyte concentrations [7]. The total sweat volume available for analysis is typically low. Constituents like Na^+^, lactate and urea exhibit high molar concentrations that could potentially interfere with K^+^ detection [8]. The development of wearable sensors that can handle these constraints while providing rapid, accurate K^+^ analysis with minimal user intervention is therefore needed.

Traditional laboratory techniques for sweat testing like flame photometry, ion chromatography and inductively coupled plasma mass spectrometry (ICP-MS) offer high accuracy [9]. Nonetheless, they require significant sample volumes, multi-step sample handling and clean-room facilities with sophisticated instrumentation. These requirements severely restrict field-based sweat testing applications. Alternatives like colorimetric assays and pH indicators can be incorporated onto flexible substrates but provide only semi-quantitative estimates of analyte levels [10].

Recent advances in wearable biosensors and microfluidics have enabled more convenient sweat analysis capabilities. Miniaturized ion-selective electrodes fabricated on soft contact lenses, tattoo electrodes and microfluidic patches have been explored for real-time electrolyte quantification [11,12]. However, most sweat-based ion-selective electrode (ISE) sensors report Na^+^ and pH monitoring, with limited focus on K^+^ detection [13]. Moreover, transducer materials like metals, conducting polymers and carbon nanotubes suffer from limitations like drift, fouling, cost and complexity [14].

Graphene, owing to its high conductivity, chemical stability and ease of functionalization, presents as an attractive transducer material for wearable electrochemical sensing [15,16,17,18]. Previously, RGO flakes drop-casted on electrodes have shown promise for K^+^ monitoring [19]. However, dispersion and aggregation issues with RGO suspensions pose challenges. The use of cyclodextrins to functionalize graphene into printable, stable inks has been shown to overcome these limitations [20]. This paves the path for the scalable fabrication of graphene-based wearable sensors using techniques like screen printing.

In this work, we synthesize β-CD-RGO and explore its use as an ion-to-electron transducer in printed electrodes for K^+^ quantification in sweat. β-CD prevents the aggregation of RGO sheets through steric repulsion while the hydroxyl groups provide selective sites for K^+^ capture. Electrodes fabricated by screen printing β-CD-RGO on conductive carbon substrates are characterized extensively. A solid-state ion-selective membrane is integrated on the printed electrodes. The sensor’s analytical performance in terms of sensitivity, dynamic range, reproducibility and on-body sweat measurements is investigated. Interference from ions like Na^+^ and surface-active molecules is evaluated. Ultimately, this presents a facile strategy for synthesizing stable, printable RGO inks for wearable sweat sensor development. By addressing existing limitations with sweat K^+^ monitoring, this sensor could find wide applicability in non-invasive diagnostics, fitness monitoring and electrolyte imbalance detection.

## 2. Results and Discussion

**Figure 1:**
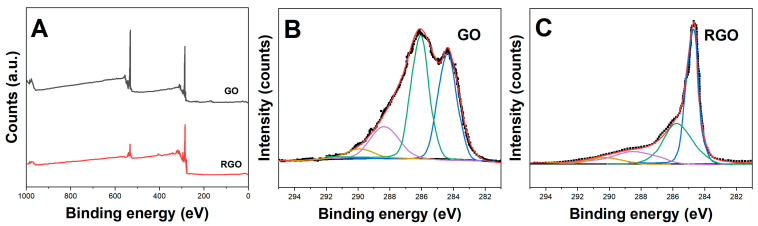
(**A**) XPS survey scans of GO and RGO. High-resolution C1s XPS spectra of (**B**) GO and (**C**) RGO.

The synthesized GO, RGO and β-CD-RGO materials were characterized using XPS, FT-IR, Raman spectroscopy and FESEM imaging. Figure 1A shows the survey scan XPS spectra of GO and RGO. The peak intensity of C_1s_ and O_1s_ signals can be used for calculating the C/O ratio. The C/O ratio increased from 2.1 for GO to 7.9 for RGO, indicating effective oxygen functional group removal during the reduction process. The high-resolution C1s spectra in Figure 1B,C reveals that GO contained high amounts of oxygen-containing functional groups like C-O (286.1 eV), C=O (287.8 eV) and O-C=O (288.5 eV) [21]. After reduction, the C-C sp^2^ peak at 284.4 eV became predominant in RGO while the oxygen functionalities reduced significantly [22].

**Figure 2:**
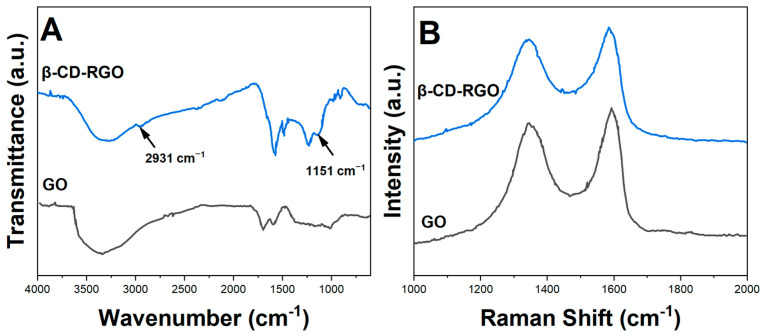
(**A**) FTIR spectra; (**B**) Raman spectra of GO and β-CD-RGO.

Figure 2A shows the FTIR spectra of GO, RGO and β-CD-RGO. The presence of various oxygen functionalities could be inferred in GO from the peaks at 1720 cm^−1^ (C=O), 1620 cm^−1^ (aromatic C=C), 1220 cm^−1^ (C-OH) and 1060 cm^−1^ (C-O) [23]. Most of these peaks diminished in RGO due to the removal of oxygen functional groups after reduction. In the β-CD-RGO hybrid, additional peaks at 2931 cm^−1^ and 1151 cm^−1^ were observed, which could be explained as the characteristic vibrations of β-CD [24]. 

Raman analysis further verified the structure of the graphene materials. As observed in Figure 2B, GO exhibited a higher ID/IG ratio of 1.15 compared with RGO (0.97) and β-CD-RGO (0.95), indicating more defects and disruption of the sp^2^ network after oxidation [25]. The subsequent removal of oxygen functionalities led to the lowering of ID/IG. However, β-CD-RGO showed slightly higher D and G peaks compared with RGO due to the presence of β-CD inducing some defects [26].

**Figure 3:**
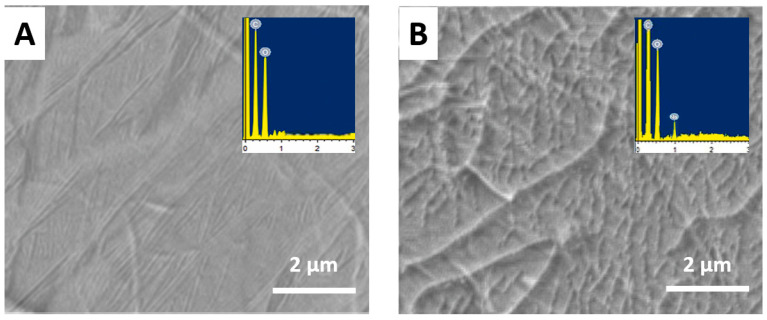
SEM images and EDAX spectra of (**A**) GO and (**B**) β-CD-RGO. Inset: EDX spectra of GO and β-CD-RGO.

The SEM images in Figure 3 revealed the morphologies of the GO sheets. GO exhibited closely stacked, crumpled sheets due to the presence of abundant oxygen functional groups. RGO sheets had a wrinkled morphology while β-CD-RGO showed the successful anchoring of β-CD molecules onto the RGO surfaces, preventing re-aggregation. Energy-dispersive X-ray spectroscopy (EDAX) confirmed the presence of elemental C and O in RGO, while an additional Na peak was observed for β-CD-RGO due to the sodium salt used for β-CD synthesis.

**Figure 4:**
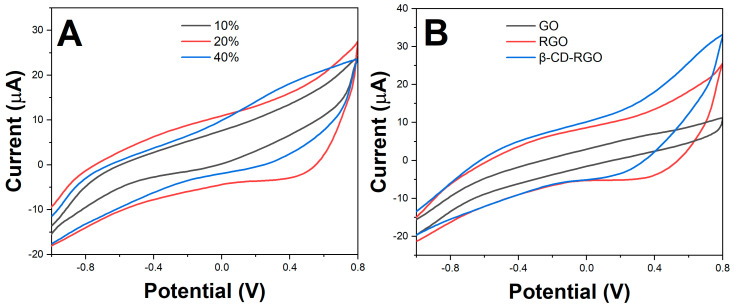
(**A**) Cyclic voltammograms of printed electrodes with different RGO loadings. (**B**) Cyclic voltammograms of printed electrodes modified with GO, RGO and β-CD-RGO.

The electrochemical properties of the printed electrodes with different RGO loadings were studied using CV. Figure 4A shows the cyclic voltammograms obtained for 10%, 20% and 40% RGO loadings in the printed electrode when tested in 1 mM KCl solution. With increasing RGO content, the voltammetric current response increased due to the greater number of electron transfer sites. However, beyond 20% RGO loading, the current reduced. This could be attributed to the increased sheet aggregation at higher loadings that lowers the effective electrochemical surface area. The 20%-RGO electrode gave an optimal response and was used for subsequent studies.

Further, the performance of the bare printed CC-ink electrode was compared with those modified with GO, RGO and β-CD-RGO. Figure 4B shows that the β-CD-RGO-modified electrodes exhibited the highest current, indicating good electrocatalytic behavior. This stemmed from the synergistic combination of high conductivity from RGO and excellent dispersibility afforded by β-CD functionalization.

**Figure 5:**
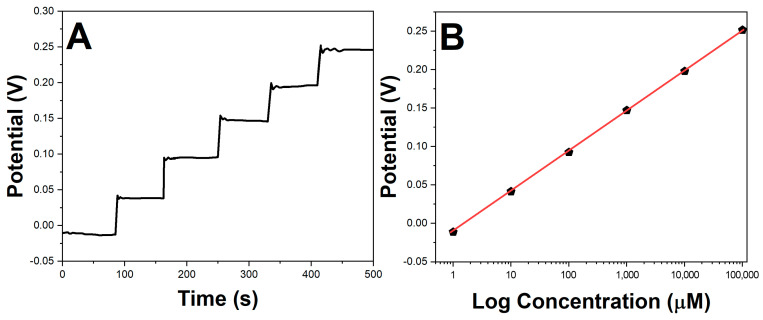
(**A**) Open circuit potential response of printed sensors with successive addition of KCl. (**B**) Calibration curve of printed potassium sensor.

The potassium response of the printed electrodes modified with β-CD-RGO and coated with a K^+^-selective membrane was evaluated via open circuit potential (OCP) measurements. Figure 5A shows the time-based OCP response of the printed sensors when sequentially dipped in KCl solutions of increasing concentration from 1 μM to 100 mM. A clear increase in potential was observed with increasing potassium levels. 

Figure 5B shows the calibration curve obtained by plotting the measured OCP versus the logarithm of KCl concentration. The printed electrodes exhibited a linear response over a wide dynamic range of 1 μM to 100 mM (R^2^ = 0.998), covering the typical potassium levels in human sweat. A slope of 57.2 mV/decade was obtained, which is close to the 59 mV/decade expected from the Nernst equation for a monovalent cation like K^+^. This indicated the high sensitivity of the printed sensors toward potassium. A low detection limit of 1 μM was observed.

The reproducibility of the printed potassium sensors was evaluated over 10 successive calibration runs and the results are shown in Table 1. The relative standard deviation (%RSD) in slope was less than 3.5%, highlighting the excellent reproducibility of the printed sensors over multiple calibration cycles. This can be attributed to the chemical stability of graphene along with the uniformity achieved during screen printing.

**Figure 6:**
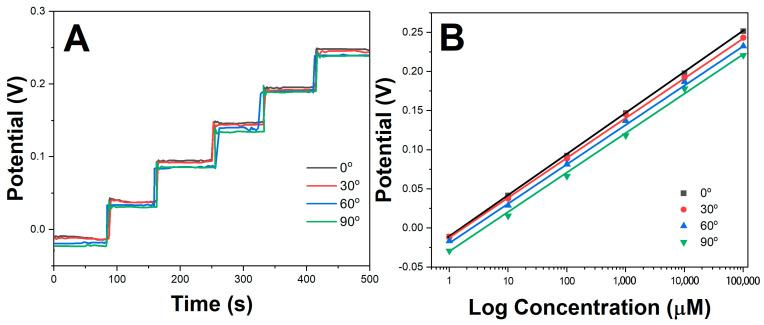
(**A**) OCP response of printed sensors under different bending angles. (**B**) Calibration curves of printed sensors under different bending angles.

The mechanical flexibility of the printed potassium sensors was studied by attaching the electrodes onto a bending test apparatus and evaluating the sensor performance at different bending angles. Figure 6A shows the stepwise OCP response of the printed sensors when bent at 0°, 30°, 60° and 90° angles and tested with successive potassium concentrations from 1 μM to 100 mM. Minimal deviation in the OCP values was observed even at 90° bending, highlighting the excellent flexibility afforded by the printed β-CD-RGO transduction layer. Figure 6B plots the calibration curves obtained under the different bending conditions. Negligible differences were observed in the sensitivity and linear response ranges, with the sensitivity varying from 57.2 mV/decade at 0° to 56.1 mV/decade at 90° bending. This demonstrated the robustness of the printed β-CD-RGO electrodes under mechanical deformation.

**Figure 7:**
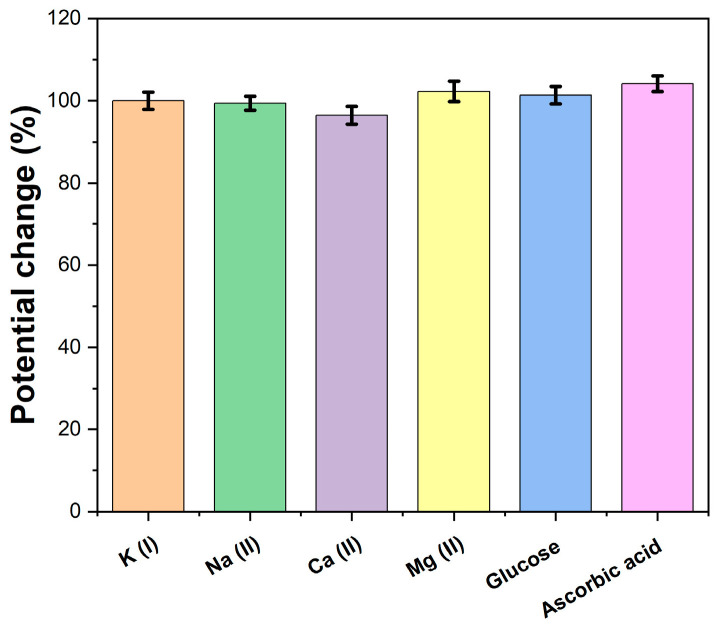
Selectivity of printed sensors against potential interferents.

The selectivity of the printed potassium sensors was studied by measuring the open circuit potential response in a 10 mM KCl solution spiked with 100 mM Na^+^, 10 mM Ca^2+^, 10 mM Mg^2+^, 10 mM glucose and 10 mM ascorbic acid as potential interferents. Figure 7 shows the relative potential response of the printed sensors when tested with KCl containing the interferents versus KCl alone. Negligible response deviations of <5% were observed, even at 10 times higher interferent concentrations. This highlighted the good selectivity of the K^+^-selective membrane along with the underlying β-CD-RGO transduction layer. The hydroxyl groups on β-CD-RGO likely provided some selective sites for potassium capture as well. Overall, the printed sensor demonstrated resistance against potential interferences present in sweat, thereby ensuring measurement reliability.

On-body sweat testing trials were conducted by placing the printed potassium sensors on a human subject’s forearm along with a commercial sweat rate sensor. Cycling exercise was performed to stimulate sweating. Once adequate sweat was visually observed on the skin under the electrodes, the sensors were removed and rinsed in deionized water. The potassium content was measured immediately by dipping the printed sensors in the collected sweat sample. For validation, the sweat sample was also analyzed using ICP-MS (Table 2).

A total of 5 on-body measurements were conducted. A linear correlation (R^2^ = 0.982) was obtained, with the values showing <10% relative deviation on average. This confirmed the ability of the printed β-CD-RGO sensor to reliably measure potassium levels in human sweat. The facile sensor fabrication and measurement approach demonstrates strong promise for non-invasive, wearable real-time sweat analysis.

## 3. Experimental Methods

### 3.1. Materials 

Graphite powder, β-CD, L-ascorbic acid, ammonium hydroxide, potassium chloride, sodium chloride, potassium tetrakis(pentafluorophenyl)borate (KTPFPB), sodium ionophore X, high molecular weight PVC, bis(2-ethylhexyl)sebacate (DOS), tetrahydrofuran (THF) and tridodecylmethylammonium chloride (TDMAC) were obtained from Sigma-Aldrich. Commercial conductive carbon ink (CC-ink) was procured from Henkel. All chemicals were analytical grade and used as received. Aqueous solutions were prepared in Milli-Q water (resistivity ~18 MΩ-cm).

### 3.2. Synthesis of GO and RGO

GO was synthesized from natural graphite flakes using a modified Hummer’s method [27]. First, 1 g of graphite and 0.5 g of sodium nitrate were added to 23 mL of concentrated H_2_SO_4_ and cooled to 0 °C in an ice bath. Then, 3 g of potassium permanganate was added gradually under constant stirring. The mixture was then heated to 35 °C and stirred for 30 min, followed by the dropwise addition of 46 mL water. After 15 min, 140 mL of warm water was added, and the solution was treated with 3 mL of 30% H_2_O_2_. The resulting mixture was filtered and washed with 5% HCl solution and then warm water until the rinse water pH became neutral. The yielded GO sheets were re-dispersed in water via gentle sonication. The oxidation of graphite to GO was achieved through a modified Hummer’s method, which uses strong oxidizing agents to introduce oxygen-containing functional groups onto the graphene sheets. This process begins with graphite flakes and sodium nitrate being added to concentrated H_2_SO_4_ and cooled to 0 °C. KMnO_4_ is then gradually added as the oxidizing agent. The reaction proceeds as follows:

3C + KMnO_4_ + 5H_2_SO_4_ → 3CO_2_ + 3CO + MnSO_4_ + K_2_SO_4_ + 5H_2_O

The KMnO_4_ oxidizes the graphite structure and is reduced to Mn^2+^ ions. This results in the graphite being converted to GO, which contains epoxy, hydroxyl, carbonyl and carboxyl groups on the graphene sheets. The addition of water results in the formation of MnO_2_ according to the reaction:

2MnSO_4_ + 2H_2_O + 1/2O_2_ → 2MnO_2_ + 2H_2_SO_4_

The MnO_2_ is subsequently removed via treatment with H_2_O_2_, which reduces it back to soluble manganese sulfate.

MnO_2_ + H_2_O_2_ → MnSO_4_ + O_2_ + H_2_O

The resulting mixture contains the GO sheets dispersed in water. This GO can then be chemically reduced to yield RGO.

RGO was prepared via the chemical reduction of GO. First, 40 mL of the as-prepared 2 mg/mL GO aqueous dispersion was diluted to 60 mL. Ammonium hydroxide was added to adjust the pH to 10. Then, 20 mg of ascorbic acid was added as the reducing agent and the solution was stirred at 95 °C for 1 h. The color of the dispersion changed from light brown to black, indicating the formation of RGO. It was then centrifuged at 6000 rpm for 20 min and the supernatant removed. The settled RGO was washed thrice with water to remove residual salts and re-dispersed in water.

The reduction of GO to RGO is a critical step that helps restore the graphene structure by removing oxygen-containing functional groups. The reduction is carried out by utilizing reducing agents that can donate electrons to the GO sheets. Here, ascorbic acid was used as the reducing agent with ammonium hydroxide to adjust the pH to 10.

The reduction reaction proceeds through the following mechanism:

First, in alkaline conditions, the ascorbic acid dissociates to generate ascorbate anions:

C_6_H_8_O_6_ + OH^−^ → C_6_H_7_O_6_^−^ + H_2_O

The ascorbate anion acts as the electron donor, giving an electron to the GO:

C_6_H_7_O_6_^−^ + GO → C_6_H_6_O_6_ + RGO

In this process, the oxygen functional groups like epoxy and hydroxyl groups on the GO are removed by the electron donation from ascorbate. This occurs through the elimination of H_2_O and formation of C=C bonds:

R-O-R + 2e^−^ + 2H^+^ → R-R + H_2_O

The carboxyl groups are reduced to hydroxyl groups:

RCOOH + 2e^−^ + 2H^+^ → RCH_2_OH

### 3.3. Fabrication of β-CD-Functionalized RGO (β-CD-RGO)

A total of 100 mg of β-CD was added to 10 mL of the 2 mg/mL RGO dispersion and sonicated for 15 min [28]. In addition, 20 μL of ammonium hydroxide was added to increase the pH to 9.5–10. The mixture was heated to 60 °C under stirring for 12 h. The final black dispersion was centrifuged at 8000 rpm for 15 min and re-dispersed in Milli-Q water. This yielded a 1 mg/mL β-CD-RGO stable aqueous ink.

The covalent functionalization of RGO with CD occurs due to the reaction between the hydroxyl groups present on CD and the carboxyl and epoxy groups found on the basal planes and edges of RGO. This reaction is facilitated by the alkaline pH and heating which promotes the nucleophilic substitution reaction.

The mechanism involves the ring opening of the epoxide groups on RGO by the hydroxyl groups on CD. This forms an ether linkage between the CD molecule and the RGO sheet. Meanwhile, the carboxyl groups on RGO can also react with the hydroxyls on CD to form ester linkages. These reactions result in stable covalent attachment of CD molecules onto the RGO nanosheets. The CD-functionalized RGO contains hydrophilic cyclodextrin groups on its surface, which enhances its water dispersibility and prevents re-aggregation of the RGO nanosheets. This allows for the preparation of stable aqueous dispersions of CD-RGO.

### 3.4. Material Characterization

The synthesized GO, RGO and β-CD-RGO were characterized via FT-IR spectroscopy (Bruker Alpha II, Billerica, MA, USA) and Raman spectroscopy (HORIBA XploRA, Kyoto, Japan) for structural and vibrational analysis. To obtain accurate ID/IG values, we first baseline-corrected the spectra by subtracting a linear fit to the background signal. We then used multi-peak fitting with Lorentzian peak shapes to deconvolve the distinct D and G bands. The peak positions were constrained based on expected wavenumbers, but the peak widths and intensities were allowed to vary during fitting. X-ray photoelectron spectroscopy (Kratos AXIS Supra, Manchester, UK) was used to analyze surface elemental composition and chemical states. Morphology was examined under field emission scanning electron microscopy (FESEM, ZEISS Sigma, White Plains, NY, USA).

### 3.5. Electrode Fabrication

Screen-printable electrodes were fabricated on glossy paper substrates using a semi-automatic screen printer (AMI MSP485). The CC-ink was printed as the conductive layer and thermally cured at 120 °C for 1 h. β-CD-RGO ink was then printed on top of the CC-ink layer to serve as the transducer. A solid-state reference electrode was fabricated by overprinting the CC-ink with silver chloride and potassium chloride inks. The printed electrodes were cut into 4 mm diameter discs using a laser cutter.

### 3.6. ISE Preparation and Drop Casting

The ISE cocktail was formulated by dissolving 250 mg PVC, 66 mg DOS, 3 mg sodium ionophore X and 1 mg KTPFPB in 5 mL THF [29]. For the K^+^-ISE, valinomycin was used instead of the sodium ionophore. The ISE cocktail was drop-cast on the β-CD-RGO printed electrodes and dried overnight before sensor testing.

### 3.7. Electrochemical Measurements

CV was performed on the printed electrodes using a potentiostat (Metrohm Autolab) in the presence of 1 mM KCl from −0.2 to 0.6 V vs. Ag/AgCl at a scan rate of 50 mV/s. This evaluated the electrochemical performance of the electrodes. For potassium response measurements, open circuit potential (OCP) was recorded by dipping the printed electrodes in KCl solutions from 10^−5^ M to 10^−1^ M and measured using a digital multimeter with high input impedance.

Sensor Optimization and Calibration. The composition of the sensing platform was optimized by varying the β-CD-RGO loading from 10% to 50% in the printed ink formulations. CVs were performed for each composition in 1 mM KCl to determine the formulation giving maximum current. Printed electrodes were calibrated over a linear range of 10 μM to 100 mM KCl by measuring the OCP. The slope, linearity, limit of detection (LOD) and sensitivity were determined.

### 3.8. Reproducibility, Bending Tests and Interference Studies

The reproducibility of the printed potassium sensors was evaluated by performing measurements in 10 mM KCl over 10 cycles. Bending tests were carried out by attaching the printed electrodes to a mechanical bending setup and characterizing the sensor response at different bending angles. Potential interferences were studied by measuring the potassium response (10 mM KCl) in the presence of 100 mM Na^+^, 10 mM Ca^2+^, 10 mM Mg^2+^, 10 mM glucose and 10 mM ascorbic acid.

## 4. Conclusions

In summary, a wearable sweat sensor based on screen-printed β-CD-RGO functionalized with β-CD was developed for the real-time monitoring of potassium levels. The β-CD-RGO composite provided optimal electrochemical performance as an ion-to-electron transducer by preventing the aggregation of RGO sheets. Electrodes fabricated by screen printing β-CD-RGO on conductive carbon substrates exhibited excellent flexibility and electrocatalytic behavior. The integration of a solid-state potassium ion-selective membrane resulted in the printed sensors demonstrating a wide linear range (1 μM–100 mM), low detection limit (1 μM), high sensitivity (57.2 mV/decade) and negligible interference against ions like Na^+^ and Ca^2+^. Bending tests revealed minimal effects on sensor performance even up to 90° angles. On-body trials showed <10% deviation versus ICP-MS validation, confirming the ability for accurate sweat potassium quantification. Overall, this work successfully established an effective functionalization and fabrication strategy for synthesizing printable β-CD-RGO inks to develop flexible, disposable, wearable sweat sensors. With further optimization and validation, such sensors could find diverse applications in non-invasive electrolyte monitoring, sports performance tracking, cystic fibrosis diagnosis and kidney disorder assessment.

## Figures and Tables

**Table 1:** Reproducibility of printed potassium sensor over 10 calibration runs.

Run No.	Slope (mV/decade)	Run No.	Slope (mV/decade)
1	56.8	6	57.2
2	57.7	7	56.5
3	58.3	8	58.1
4	56.4	9	55.6
5	55.9	10	57.8
Average	57.1	RSD	3.4%

**Table 2:** Comparison of potassium concentration values obtained from printed sensor and ICP-MS for on-body sweat testing.

Sample No.	Printed Sensor K^+^ Concentration (mM)	ICP-MS K^+^ Concentration (mM)	% Relative Deviation
1	12.5	13.2	5.3
2	16.8	15.9	5.7
3	20.3	22.1	8.1
4	18.2	19.5	6.7
5	14.6	13.8	5.8
Average	16.5	16.9	6.3

## Data Availability

The data that support the findings of this study are available from the corresponding author upon reasonable request.
